# The Climate of Florida

**Published:** 1882-11-20

**Authors:** James C. Neal


### THE CLIMATE OF FLORIDA.

By James C. Neal, M. D., Ph.C.

Dr. Haygood's experience, as given in the Record of August 12, conflicts decidedly with that of most medical men in this state. In my own case several years' residence in the highlands of Central Florida have relieved me of a harassing cough, night sweats, a hectic flush, anorexia, and from the invalid of one hundred and thirty pounds weight, dreading the cold, I now am able for continuous labor, weigh one hundred and sixty-six pounds, and have long ago discontinued the use of cod-liver oil, phosphites, etc.

Dr. Z. H. M——, of Apopka. Fla., has a similar experience. Coming from Georgia to find a temporary respite, year after year has passed away and the doctor now is the picture of rosy health in his old age.

Dr. C. J. K——, of Jacksonville, an old traveler, a scientific investigator, and widely known for his own case, has tried many climates, and has fixed upon Florida as the best.

Twelve years ago Dr. McM——, of Charleston, S. C., brought his wife, a confirmed consumptive, to Gainesville, Fla., to spend what seemed a small remnant of life more comfortably. To-day she bids fair to reach the "three score and ten," despite the "exceedingly debilitating" climate.

Col. J. H. R——. came from South Carolina as a winter resort, having all the symptoms of phthisis in an advanced stage. Now he is an active, comparatively healthy man, utterly unlike his former self.

I need not multiply examples, almost every neighborhood in this section can supply them, of the competent medical men who in their own cases are satisfied of the value of this climate as both preventive and curative of tuberculosis.

In this section of the State, high, dry, rolling pine land, and perfect drainage, in six years and in one thousand cases I have seen but four of pneumonia, and two of these traumatic. Can Dr. Haygood say as much even for Beaufort or Raleigh?

During the summer we have a mild form of malaria, but as to effect of such a climate upon phthisis I would refer him to Dr. Lawson, pp. 297, 388. Aitken, vol. i., p. 265.

These malarial attacks are easily subdued, and fatal cases are rare. Too many physicians allow their patients to remain in a cold, changeable climate until the lungs are a mass of tubercle, then send them to Florida too feeble to take out-door exercise, often with but very little money to procure attentions or delicacies necessary, and among strangers the poor invalid becomes nostalgic and sinks rapidly, and then Florida is blamed. This ought not to be, but every season finds our hotels filled with cases that should be at home among friends, making ready for the rapidly approaching close of life.

There is, however, a positive curative agency in this climate for certain ailments, and to guide the profession, I beg to submit the results of many years of careful study both of cases and climate factors.

These factors are location, soil, drainage, water, sunshine, temperature, humidity, and air-pressure. And with reference to a strip of elevated land, lying forty miles from either coast and extending from 28.30° to 30° latitude, I think no place east of the Rocky Mountains is better suited for the amelioration if not cure of incipient tuberculosis, asthma, catarrh, nervous complaints and bronchitis.

*The location*—Almost insular, constantly traversed by warm breezes from the sea and gulf, in the track of trade winds, and the freshness tempered by miles of almost unbroken forest.

*Soil*—Very sandy with but little humus, or animal remains; configuration broken, rotting, often eighty to one hundred and fifty feet elevation above the gulf. Drainage perfect, no stagnant pools or marshes.

*Water*—In the main freestone, in wells twenty-five to sixty feet deep, with an average temperature of 65° F.

*Timber.*—The long-leafed pine, with little undergrowth, allowing free circulation of air.

*Sunshine.*—Twenty-two days of each month of almost clear skies, the remainder often partly available for out-door exercise, and during the winter the rains are usually at night, leaving the day cool and bright (see note.) The rainy season begins in June and lasts till September. Often a week of showery days, then clear skies a week. During the rains the temperature falls to 71° F., and this season, in comparison with the torrid days of the north, quite endurable, and as these showers rarely last but a few hours, the remainder of the day is available for exercise or labor.

*Temperature.*—Unquestionably a climate allowing a loose easy costume with few wraps, and no requirements of hot, stove-heated, badly ventilated rooms, is best for weak lungs. The houses of this section are built for free ventilation—no stagnant air in cellars or close dirty bedrooms. Blizzards, northers, sudden changes, causing cold extremities, visceral congestions, or surface chilling, are extremely rare, and as they occur, if at all, in December or January, can easily be kept from harming.

The summer maximum is usually 96° F., and this for only a few hours. But 96° F. is not common. In 1880 it reached that point but four times in June, once in July, and once in August; this year twice in July and once in August.

The night temperature for the months from May to October is from 70° to 76°, and from October to May, 60° to 65°. The hot, sweltering nights so common in the North are unknown here. October, November, and till April, are months comparable with the Indian summers of Pennsylvania—days dry, breezy and bracing, the whole season enjoyable. (See Note.)

*Humidity.*—Much has been said of the dampness of Florida, but the observations of the Signal Service show nothing excessive in its humidity, and this section compares favorably with most health resorts, the annual mean for a long series of years being 68° against 73° at Menton, 68° at Nassau, 67° at St. Paul, Minn.

*Air-pressure.*—I have no doubt but that this is the most important factor of value in our climate—its remarkable equability of air-pressure. If we regard in the lung the pressure of the heart upon the mass of the blood as balanced against the air-pressure and strength of the blood-vessel walls, it is readily seen that in a diseased lung, a decrease of air-pressure tends to congestion, and at points of weakened tissues from any cause, if the air-pressure be sensibly diminished, hemorrhage may occur. Any physician will recall instances showing that the changeable months of spring and autumn occasion great distress to tuberculous patients, and the cold of January was not as fatal as the storms of March. A fall of even one-tenth inch mercury in the barometer produces a decided discomfort, and the descent of half an inch a temporary congestion, dyspnœa, headache, or neuralgic pains. Hence patients can often predict storms from their disagreeable sensation.

A climate offering the maximum of air-pressure and the minimum of change, with a genial temperature, leaves but little to be desired for patients requiring rest and warmth, and this section surely meets the wants of such cases. (See Note.)

A dozen invalids live near me, some hemorrhagic, some neuralgic, but all have experienced relief, and the majority of cases of rheumatism, asthma, bronchitis and catarrh are alike favorably affected by this equability.

The practical deductions are readily apparent.

Northern invalids, in which the deposit of tubercle is incipient or limited; aged invalids requiring rest, warmth and relaxation; neuralgic cases, nerve-worn patients, the whole range of uterine troubles resulting from congestions, all cases that grow better in the warm settled days of May or October, and get worse in November and March—these should be advised to try Florida, especially this section, with hope of betterment.

With discharging cavities, or fully tubercularized lungs, little aid can be given, though our 220 clear days, average air-pressure of 30.03 inches, and mean temperature of 71° will aid in pleasantly prolonging life.

Many when here neglect the most common sanitary laws, expose themselves recklessly to heat, rain and dew, take fatiguing exercise, sit in draughts, eat to excess, and do not derive the benefit they should.

A little common sense would obviate this, and save much blame now given to Florida.—*N. Y. Med. Rec.*

---

NOTES.—*Temperature.* Mean, December and January, 55°; February, 58°; March and November, 63°; April and October, 70°; May and September, 77°; June, 80°; July and August, 82°.

